# Assessing the accuracy of altitude estimates in avian biologging devices

**DOI:** 10.1371/journal.pone.0276098

**Published:** 2022-10-26

**Authors:** Kimberly A. Lato, Julia E. F. Stepanuk, Eleanor I. Heywood, Melinda G. Conners, Lesley H. Thorne

**Affiliations:** 1 School of Marine and Atmospheric Sciences, Stony Brook University, Stony Brook, New York, United States of America; 2 Department of Ecology and Evolution, Stony Brook University, Stony Brook, New York, United States of America; San Diego Zoo Institute for Conservation Research, UNITED STATES

## Abstract

Advances in animal biologging technologies have greatly improved our understanding of animal movement and distribution, particularly for highly mobile species that travel across vast spatial scales. Assessing the accuracy of these devices is critical to drawing appropriate conclusions from resulting data. While understanding the vertical dimension of movements is key to assessing habitat use and behavior in aerial species, previous studies have primarily focused on assessing the accuracy of biologging devices in the horizontal plane with far less emphasis placed on the vertical plane. Here we use an Unaccompanied Aircraft System (UAS) outfitted with a laser altimeter to broadly assess the accuracy of altitude estimates of three commonly used avian biologging devices during three field trials: stationary flights, continuous horizontal movements, and continuous vertical movements. We found that the device measuring barometric pressure consistently provided the most accurate altitude estimates (mean error of 1.57m) and effectively captured finer-scale vertical movements. Conversely, devices that relied upon GPS triangulation to estimate altitude typically overestimated altitude during horizontal movements (mean error of 6.5m or 40.96m) and underestimated amplitude during vertical movements. Additional factors thought to impact device accuracy, including Horizontal- and Position- Dilution of Precision and the time intervals over which altitude estimates were assessed, did not have notable effects on results in our analyses. Reported accuracy values for different devices may be useful in future studies of aerial species’ behavior relative to vertical obstacles such as wind turbines. Our results suggest that studies seeking to quantify altitude of aerial species should prioritize pressure-based measurements, which provide sufficient resolution for examining broad and some fine-scale behaviors. This work highlights the importance of considering and accounting for error in altitude measurements during avian studies relative to the scale of data needed to address particular scientific questions.

## Introduction

Over the last two decades, advances in animal biologging technologies have greatly improved our understanding of animal distribution and behavior over both large and small spatiotemporal scales [1, 2]. In particular, the development of lightweight data loggers and tracking devices has improved our ability to study animals that are highly mobile but require devices with minimal weight and/or drag so as to minimize inhibiting their movements [3–8]. However, biologging devices use different methods for estimating spatial positions through time and thus have varying degrees of accuracy, which can greatly impact the resolution of data and the ability to draw certain conclusions across studies [9]. For example, a recent study suggests that Global Location Sensing (GLS) devices, which use light levels to estimate location, have a mean positional error of approximately 300km in the horizontal dimension [10]. Global Positioning System (GPS) tracking devices that use satellites to estimate locations can vary considerably in their positional error, with as little as 5m error in some devices [11] and up to 100m error in others [12]. Animals that fly or swim show considerable movement in both the vertical and horizontal planes, and thus accurately estimating their position and movement in both planes is critical for understanding their movements and behavior. For diving animals, measurements of pressure underwater can provide accurate depth estimates since pressure changes much more rapidly with depth than with altitude and the accuracy of pressure-based depth estimates have been thoroughly assessed for a range of devices [13, 14]. In contrast, the accuracy of biologging device-based altitude estimates has received little attention to date despite their increased use in studying vertical habitat use and behavior of aerial species [15–17].

Studying vertical movements can help identify vertical niche space in aerial species [18] and can provide insights into behavioral states and dynamic flight movements [16, 19]. It can also improve our understanding of changes in the behavior of aerial species relative to environmental variables, such as wind, topography, and thermals [20–23], or to the presence of human-made structures [17, 24]. The recent and ongoing development of wind farms in different regions around the world has highlighted an urgent need to understand vertical habitat use of avian species relative to the height of wind turbines (e.g. Corman & Garthe 2014, Cleasby et al. 2015). Much concern has arisen with respect to ecological implications of wind farms on avian species due to their ability to spatially displace migratory and foraging groups and cause lethal collision [25–28], which could put vulnerable species further at risk. Thus, there is a strong need to obtain accurate flight altitude estimates of aerial species to not only improve ecological studies, but also to inform management practices and impact assessments of wind farms.

Avian biologging devices typically use one of two techniques for estimating flight altitude: GPS triangulation or measurements of barometric pressure. Various factors that could impact the accuracy of the altitude measurements of these sampling techniques have been theorized [29], though we are not aware of another study that has quantitatively assessed the accuracy of flight altitude estimates of different tracking technologies due to the difficulty in obtaining true altitude estimates in real-time for comparison. Here, we deployed an array of biologging devices attached to an Unaccompanied Aerial System (UAS) outfitted with a laser altimeter to provide continuous and accurate measurements of flight height against which to compare altitude estimates from biologging devices. Our specific objectives were to: 1) quantify the accuracy of altitude estimates across multiple avian biologging devices that use either GPS triangulation or barometric pressure sampling to estimate altitude; 2) assess the accuracy of altitude estimates while stationary and moving in horizontal and vertical dimensions, respectively; and 3) assess the effects of additional factors thought to impact GPS device accuracy, such as the time since deployment and the Horizontal- and Position- Dilution of Precision (HDOP; PDOP), and barometric pressure device accuracy, such as air temperature and relative humidity. In doing so, we provide insight into the types of devices that are most suited for vertical tracking studies and discuss factors that could affect device accuracy.

## Methods

### Data collection

Stony Brook University (Long Island, United States) granted permission to use the Research Park facility where data collection took place. All UAS flights were conducted under Federal Aviation Administration Small UAS Rule (Part 107).

We assessed the accuracy of the following three biologging devices which are frequently used to track bird movement and behavior: AxyAir (Technosmart, Europe), CatLog Generation 2 (Catnip Technologies, Hong Kong,), and OrniTrack-25 (Ornitela, Lithuania). While all three devices provide altitude estimates, the CatLog and OrniTrack-25 devices are primarily used to obtain GPS tracks of birds and the AxyAir device provides 3D accelerometer data used to assess flight behavior and kinematics. CatLog and AxyAir devices are archival and must be recovered to obtain collected data, while OrniTrack-25 devices transmit data through the cellular network. The AxyAir device uses a barometric pressure sensor to estimate flight altitude while both CatLog and OrniTrack-25 use GPS triangulation, though post-processing algorithms of GPS-derived altitude estimates differ between these two devices and are discussed in more detail below.

All devices were simultaneously attached to a custom-built platform attached to a DJI Phantom 4 Pro+, UAS (S1 Fig), which was also outfitted with a laser altimeter (SF11/C LiDAR) according to methods described in Dawson et al. 2017. While the use of laser altimeters is infeasible in animal tracking studies due to the size and weight of these devices, these devices can provide reliable measurements of altitude against which altitude estimates from tracking devices can be compared; in field tests, this laser altimeter has been found to provide an accuracy of 99.9% at altitudes of up to 120m over land and 40m over water [30], which is substantially more accurate than the internal UAS GPS or barometric pressure-based altimeters [31]. Thus, the use of a laser altimeter was central to our goal of assessing flight accuracy as it provides an accurate and continuous measurement of flight height at high temporal resolution (1 second for our field studies) against which altitude estimates of biologging devices could be compared. To account for tilt angle associated with UAS movement, we corrected all laser altimeter altitude measurements according to Dawson et al. 2017. All biologging devices were programmed to sample every 5 seconds. Although all devices can be programed to sample at a finer resolution, 5 seconds was chosen as the sampling rate based on the tradeoff between the maximum sampling rate possible and tag battery life for the OrniTrack-25 device, which includes a solar panel to recharge the battery and is the most limited in its short-term battery life compared to the other devices. Although the CatLog device was programmed to sample at 5 second intervals, as like the other two devices, upon data review we found that the data points were only recorded every 10–20 seconds inconsistently.

**Fig 1:**
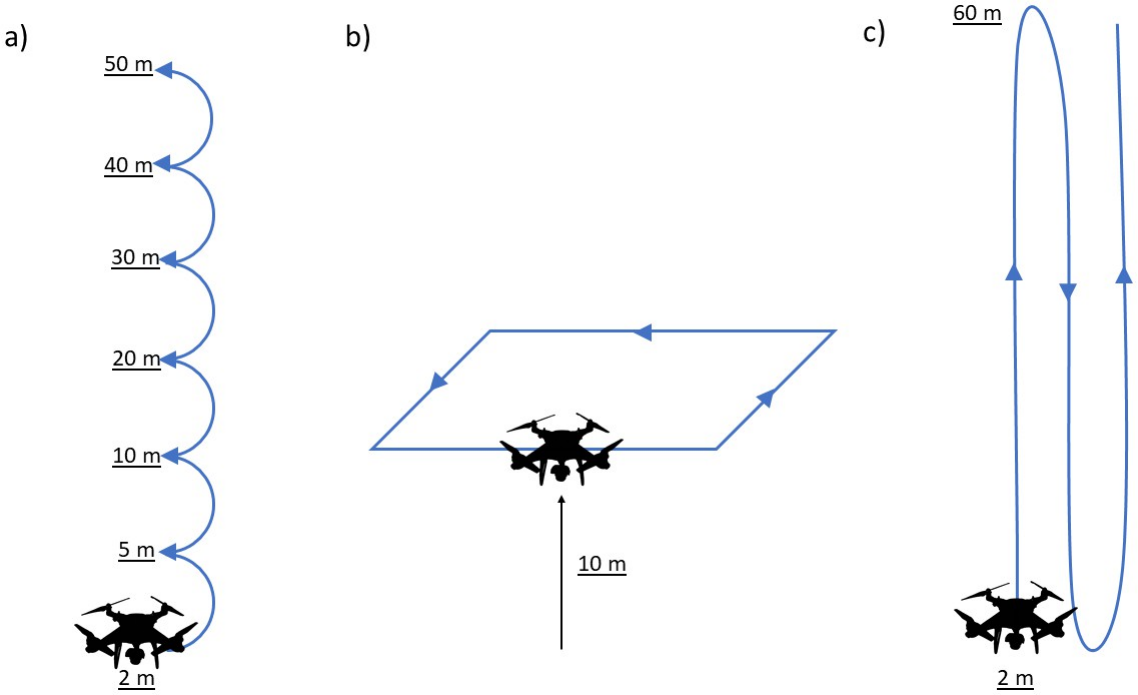
Schematic of the three field trials which used a UAS to assess the accuracy of altitude estimates of avian tracking devices. a) stationary flights performed by stopping for 30 seconds at 2, 5, 10, 20, 30, 40 and 50-meter heights; b) continuous horizontal movements where the UAS was kept at a consistent 10-meter height while moving around the perimeter of the study site (approximately 180m x 60m); c) vertical movements where the UAS made continuous ascensions/descensions from 2 to 60 meters but did not move in the horizontal plane.

We sought to examine how the altitude estimates of tracking devices was influenced by stationary, horizontal movement, and vertical movement across different altitudes and therefore conducted three types of field trials to assess accuracy (in meters above ground; Fig 1): 1) *Stationary estimates* where the UAS hovered for 30 seconds at altitudes of approximately 2, 5, 10, 20, 30, 40, and 50m. This field trial was designed to capture the accuracy of flight altitude estimates at different flight heights in the absence of effects of movement in either vertical or horizontal planes. 2) *Estimates during horizontal movements* where the UAS flew continuously at a 10m height with movement in the horizontal plane only. This trial was designed to capture the accuracy of each device’s flight altitude estimates based on the notion that GPS devices tend to increase in both accuracy and precision while moving [32, 33]. 3) *Estimates during vertical movements* where the UAS flew with continuous vertical movements (ascensions and descensions, representing movement in the vertical plane only) from 2-60m above ground. This field trial was designed to assess the ability of each device to capture continuous dynamic vertical movements, such as those performed by birds.

We used the same individual device units across all field trials except for the CatLog device which was switched for another unit after the first three flights of horizontal and first two flights of vertical field trials (the same unit was used for all flights of the stationary field trial) because it was needed in another application. To assess whether change in devices influenced results, we performed a Wilcoxon rank-sum test to determine whether the altitude accuracy in horizontal and vertical field trials was different between CatLog units. Results from this test suggested that there was no significant difference between the devices’ accuracy (measured as difference from the laser altimeter; p = 0.38), and we therefore did not differentiate between units in our results. This was the only scenario in our field tests in which a different unit was used (i.e. the same AxyAir and OrniTrack-25 units were used for all flights).

We conducted a total of 10 flights for each field trial (30 flights total). Stationary flights lasted between 4.5 and 6 minutes with an average flight duration of 5.03 minutes (± 0.43 min). Vertical flights lasted between 3 and 7 minutes with an average flight duration of 6.13 minutes (± 1.27 min). Horizontal flight trials lasted between 5 and 10 minutes, with an average flight duration of 7.28 minutes (± 1.4 min). Both horizontal and vertical flight speeds were kept between 1 and 3 meters per second. The duration of flights during field tests (total flight durations of 11–17 minutes) was determined by the battery life of the DJI Phantom 4 Pro+, which was limited by weather conditions, the additional battery draw of the SF11/C laser altimeter, and the additional weight of the biologging devices.

UAS flights for each of the three field trials were performed on different days over a period of 3.5 months to capture variability in environmental conditions (see S1 Table) and factors which might influence the performance of GPS devices, such as variability in the number of satellites connections, and the Horizontal- and Position- Dilution of Precision values (HDOP and PDOP, respectively; describing the geometric distribution of satellites in the sky), as well as factors that may influence pressure-based altitude estimates such as air temperature and relative humidity. While the variability of performance between individual units of the same device type is also a potential factor affecting the accuracy of altitude estimates in some cases (see Discussion), we were unable to assess this metric across all three tracking devices due to limitations in the number of units/devices that could be deployed during a flight; we found that increasing the weight of the custom-built platform by including additional units/devices caused the UAS battery to drain too quickly and attaching additional units would have also required a longer platform than was feasible to deploy due to complications with platform vibration and movement observed while the UAS was in flight during preliminary flight tests in which a longer platform was used.

### Flight altitude estimate

Each device required post-processing to obtain altitude estimates above ground level. As the AxyAir samples barometric pressure rather than altitude directly, pressure measurements were converted to altitude estimates in meters using a rearrangement of the formula presented in Sjöberg et al. (2018) [34]:

$$h_{i}=\frac{\left({\left(\frac{p_{0}}{p_{i}}\right)}^{\frac{1}{5.2572}}-1\right)\left(T_{i}+273.15\right)}{0.0065}$$

where *h*_*i*_ is the altitude at data point *i* in meters, *p*_*i*_ is the pressure at data point *i* in millibars as measured by the AxyAir, *T*_*i*_ is the temperature at data point *i* in degrees Celsius as measured by the AxyAir, and *p*_0_ is the pressure at ground level as measured by the AxyAir.

**Fig 2:**
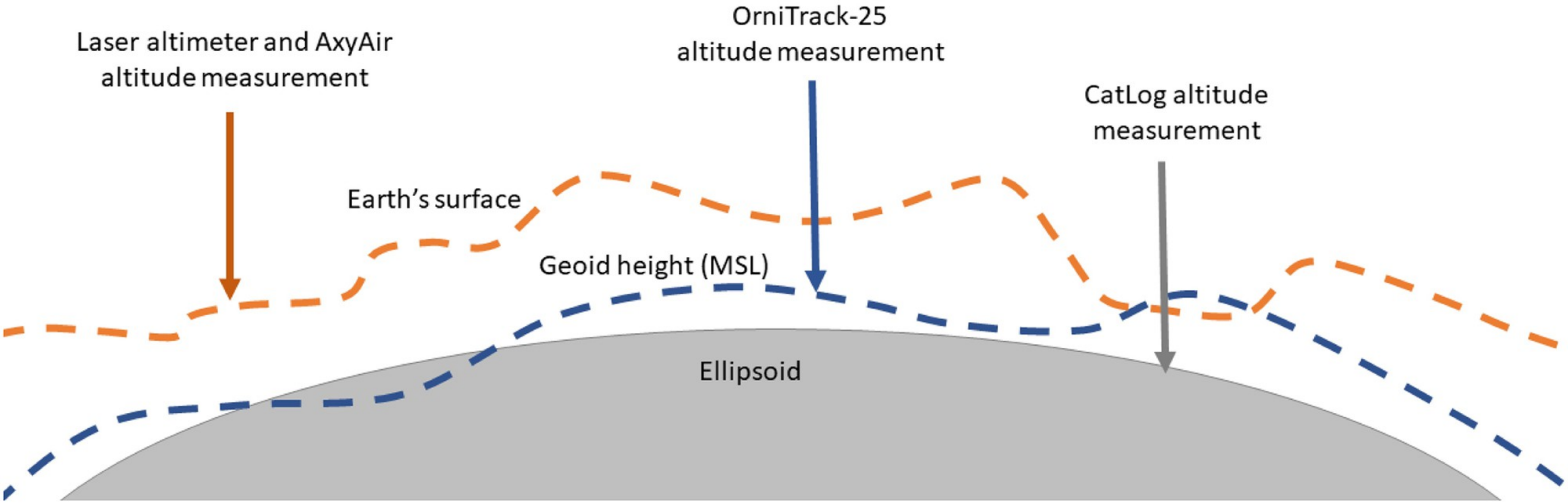
Illustration of ellipsoid, geoid (Means Sea Level; MSL), and Earth’s surface (i.e. terrain) including each device’s method of measurement and reference surface. We wanted to compare all altitude measurements relative to ground level (Earth’s surface in this figure), therefore the CatLog and OrniTrack-25 devices had to be adjusted post-hoc based on their reference surface.

The CatLog device provides the user with an altitude estimate as a height above the ellipsoid (a smoothed geographical model projection of Earth’s surface) rather than height above ground level. Thus, CatLog altitude estimates were corrected by subtracting the geoid height (a geographical model of Earth’s surface based on differences in gravitational pull due to density across Earth’s surface; -30.652m) [35] and elevation above the geoid of our study site (51.45m) [36] from raw altitude estimates (Fig 2). The OrniTrack-25 device corrects for the height of the geoid above the ellipsoid using its own internal calculations and provides the user with a height above mean sea level (MSL). Because height above MSL is approximately equal to geoid height above the ellipsoid, OrniTrack-25 altitude estimates were only corrected for elevation of our study site (51.45m above MSL) to obtain altitude estimates above ground level.

### Statistical analyses

For each metric assessed during each field trial, the normality of data was assessed statistically using a Shapiro-Wilk test and visually using a quantile-quantile (Q-Q) plot. We used either a parametric (paired t-test) or non-parametric test (Wilcoxon signed rank test) for comparative statistical analyses for normally and non-normally distributed data, respectively.

#### Stationary estimates

For each flight height (n = 10), we averaged the altitude estimate across all data points for each device type within the 30 second time interval that the UAS was hovering. We then assessed the accuracy of these estimates by comparing the average altitude estimate of each device to the average estimate of the laser altimeter during each 30 second time interval using a Wilcoxon signed rank test with flight number as the paired value.

We also assessed the relationship between DOP values and accuracy for both CatLog and OrniTrack-25. HDOP and PDOP values are often used as an indicator of 2D and 3D positional accuracy, respectively [37, 38]. Since data points with DOP values closest to zero are considered the most accurate, removing data points with high DOP values is often used as a data filtering method to increase the overall accuracy and precision of a collected dataset [37]. Although HDOP values are used as a measure of horizontal accuracy, horizontal error can insight vertical errors [29] and thus was a seemingly important consideration in our study. HDOP values <2 are often considered the most accurate in their location estimates [39, 40]; therefore, we removed all HDOP values greater than 2 and re-performed a Wilcoxon signed-rank test to assess whether the accuracy of the CatLog and OrniTrack-25 altitude estimates improved. PDOP values were not available for the CatLog and OrniTrack-25 devices during stationary tests and therefore, we could not evaluate this metric for this field trial. In subsequent field tests during horizontal and vertical movements, a different CatLog unit of the same model was used (further explanation in Data Collection section of Methods) that did provide PDOP values.

#### Estimates of altitude during horizontal movements

For flights in which we only moved in the horizontal plane (holding altitude constant at 10m; n = 10), we first assessed the average altitude estimate of each device across the entire duration of the flight. As in the analysis of stationary estimates, we performed a paired Wilcoxson signed rank test comparing the mean altitude estimate of all devices to the mean estimate of the laser altimeter using flight number as a paired value.

We also assessed whether the accuracy of altitude estimates for each device type changed with respect to variables that are thought to impact altitude estimates, specifically for devices using GPS triangulation. Following the assumption that GPS accuracy increases with connection time to a satellite due to the dependency of GPS fixes on the previous GPS fix [41], we assessed the change in each device’s accuracy through time using a linear mixed effects model with accuracy as the dependent variable, time since start of deployment as the independent variable, and flight number as a random variable using the ‘lmerTest’ package in R [42]. Additionally, we used the data collected during horizontal movements to assess whether binning the data over different time intervals impacted each device’s accuracy. Here, we averaged the altitude estimates of data points for each device within each flight into the following time intervals: 15 sec, 30 sec, 60 sec, 2 min, 3 min, 4 min. We then quantified the difference between the altitude estimates from the laser altimeter and each biologging device and averaged these differences across each flight. We performed a one-sample Wilcoxon signed rank test to determine whether these mean differences differed from zero. We also broadly assessed the relationship between the number of satellite connections and accuracy, following the assumption that accuracy typically improves with an increase in the number of satellites [43].

Lastly, as in the stationary test, we compared results of the horizontal movement field trials using data points with HDOP values <2 with all data points, respectively for both the CatLog and OrniTrack-25 devices. PDOP values were available for seven of the ten horizonal flights for the CatLog device only (OrniTrack-25 does not provide PDOP values) due to the change in the CatLog unit used, as described above. PDOP values <2 are often considered “the most accurate” [44]; therefore, we removed all PDOP values >2 from the last seven flights of our horizontal movement data and re-compared these altitude estimates to the laser altimeter.

We used hourly air temperature and relative humidity data provided by New York State Standard Mesonet [45; http://www.nysmesonet.org/] to perform a linear regression assessing the relationship between the accuracy of the AxyAir device, measured as the difference in altitude measured from the laser altimeter, and air temperature and relative humidity.

#### Estimates of altitude during vertical movements

For this field trial (n = 10), we performed continuous vertical movements while stationary in the horizontal plane to visually assess the ability of each device to capture patterns of ascension and descension (sinusoidal movement). We quantitatively assessed the mean differences in amplitude (here, measured as the height from peak to trough), altitude estimate of the wave peak, and wave period (time between wave peaks; S2 Fig) captured by each device and the laser altimeter for each flight and used a one-sample t-test to assess whether mean differences from the laser altimeter differed from zero. To assess the accuracy of each device to capture changes in altitude with respect to time, we identified the point in time when the wave peak occurred and calculated the time difference (if any) between the laser altimeter and each device with respect to those maximum altitudes.

Lastly, we assessed the effect of removing datapoints with HDOP values >2 on device accuracy for both CatLog and OrniTrack-25 devices. As like the horizontal movement tests, PDOP values were only available for 8 of the 10 vertical movement flights due to the change in CatLog unit. Again, we assessed the effect of removing datapoints with PDOP values >2 on CatLog device accuracy for the 8 flights in which PDOP values were available. All statistical analyses for all three field trials were performed using R Statistical Software [46].

## Results

We conducted UAS flights over the course of 3.5 months across a wide range of values in air temperature, cloud cover, relative humidity, wind speeds, and wind directions. More specifically, air temperatures ranged from 6.8 to 30.3°C (mean = 65.4 ± 12.2; S1 Table) and relative humidity ranged from 44.7% to 82.3% (mean = 15.3 ± 6.0; S1 Table).

### Altitude estimates during stationary estimates

**Fig 3:**
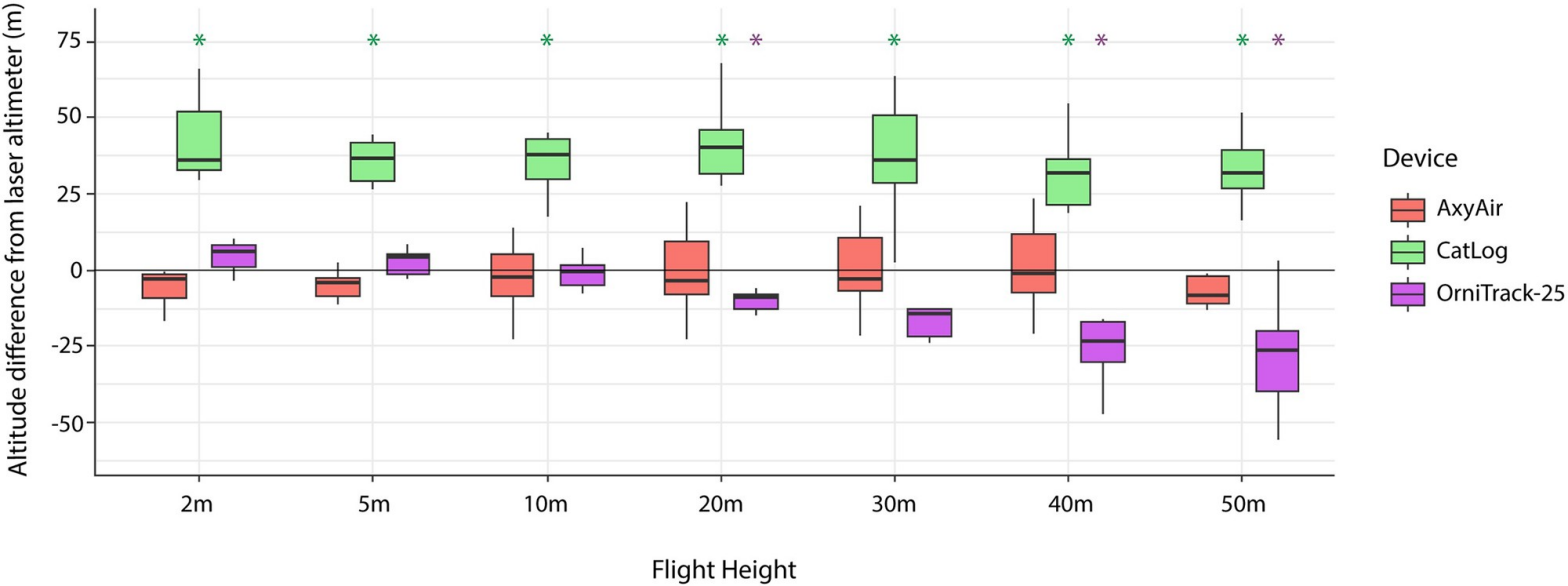
Mean difference in altitude estimates between each device and the laser altimeter measurement for the seven flight heights tested during stationary estimates. Asterisks represent significant differences from the laser altimeter measurement (α = 0.05). The black horizontal line represents zero, or no difference between the altitude estimate of the device and the measurement from the laser altimeter. As the mean difference from laser altimeter did not improve with HDOP <2, we did not include this data in this figure.

We found that the CatLog device significantly overestimated altitude for every flight height (mean error = 54.93m) during stationary field trials (S2 Table; S3 Fig), though the degree of overestimation varied slightly between flight heights (Fig 3). The CatLog device overestimated altitude by an average of 33.96m (± 27.15) at lower flight heights (2-30m) and overestimated by 20.97m (± 15.19) at higher flight heights (40–50m). While the OrniTrack-25 device, on average, underestimated flight height by (12.96m), it did not significantly differ from the laser altimeter estimate at lower flight heights (2-10m; S2 Table) but significantly underestimated the altitude by 20.43m (± 15.06) at higher flight heights (20m, 40m, 50m). However, there was inconsistency in whether the OrniTrack-25 device over or underestimated the flight height between flights (S4 Fig). While there was no significant difference between the AxyAir and the laser altimeter estimate at any of the flight heights tested during stationary tests (S2 Table), it generally underestimated the altitude by an average of 2.14m (± 11.63; Fig 3; S5 Fig). All devices had some datapoints with extreme altitude estimates, but the CatLog device consistently had the highest number of extreme estimates and the greatest standard deviation from the mean (Fig 3). CatLog datapoints with HDOP >2 included all datapoints from 3 entire flights (F23, F25, F27) which were then removed from the analysis comparing altitude estimates of data points with HDOP <2 with those produced using data points with all HDOP values, as were 7% of the data points from remaining flights. We found that using only data points with HDOP <2 did not improve the accuracy of altitude estimates of the CatLog device (S2 Table; S6 Fig). As PDOP values were not available for the CatLog device during stationary field trials, we could not assess the relationship between PDOP and estimate accuracy for stationary field tests. All OrniTrack-25 data points on all flights had HDOP <2, therefore we did not perform a separate HDOP analysis for the OrniTrack-25 device as it would provide the same outcome as the full dataset.

### Altitude estimates during horizontal movements

**Fig 4:**
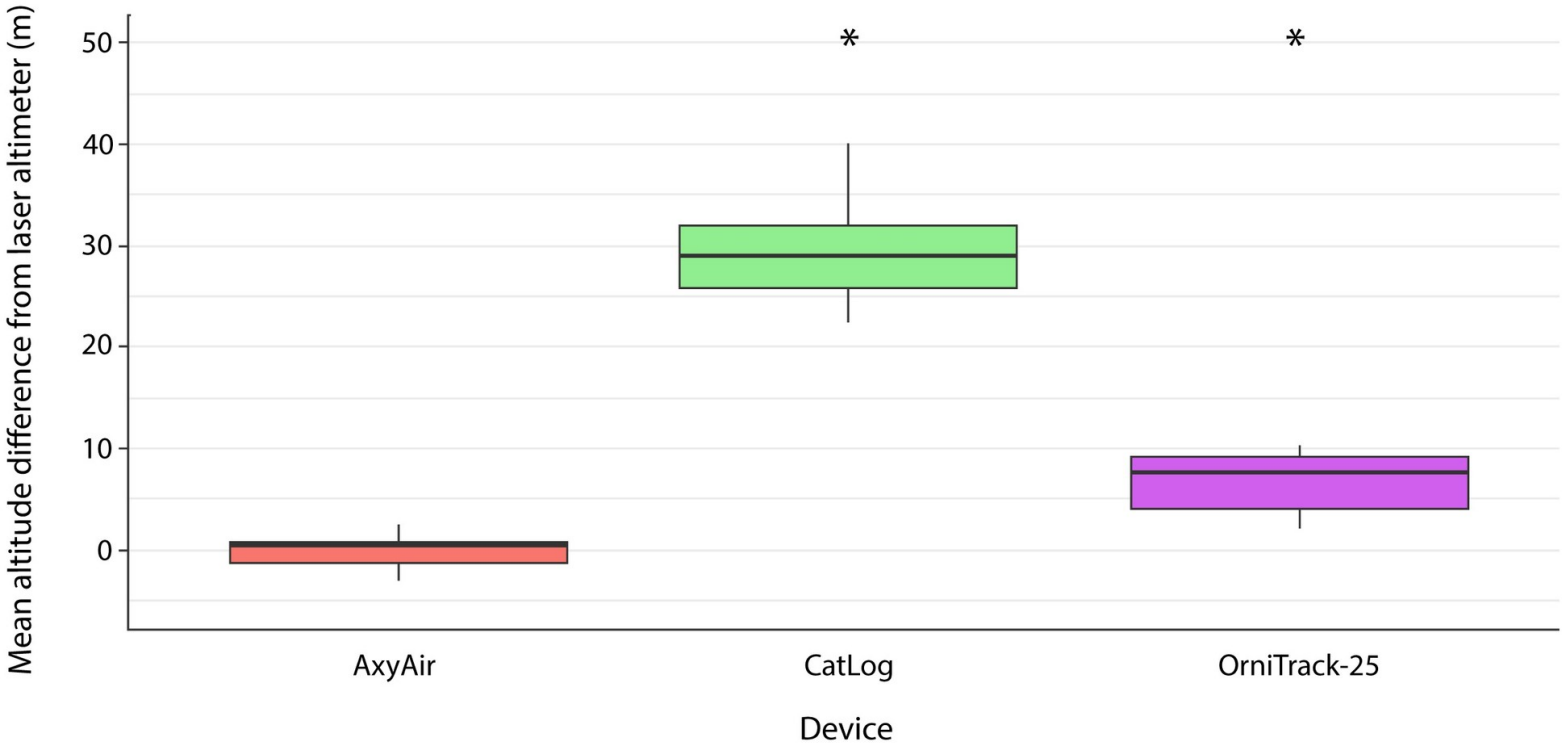
Mean difference between the altitude estimate of each device type and laser altimeter during horizontal movements. Asterisks represent significant differences from the laser altimeter (α ≤ 0.05).

During horizontal movements, both the CatLog and OrniTrack-25 devices significantly overestimated altitude (S3 Table; Fig 4) though the CatLog device overestimated altitude (mean error of 40.96 ± 29.99m) to a much greater extent than the OrniTrack-25 device (mean error of 6.5m ± 3.13). The AxyAir device tended to slightly underestimate altitude (mean error of 1.0m ± 3.35) during horizontal movements, though the difference from the laser altimeter was not statistically significant (S3 Table; Fig 4).

We found no significant linear relationship between the accuracy of altitude estimates and time since deployment for any of the devices tested during horizontal flights (S4 Table). Additionally, averaging datapoints across different time intervals did not improve the accuracy of CatLog and OrniTrack-25 altitude estimates (Table 1). However, we did find that averaging across time bins of 15 seconds resulted in a significant difference between AxyAir and laser altimeter estimates, while there was no difference when longer time intervals were used (Table 1).

**Table 1:** P-values of one-sample Wilcoxon signed-rank tests to evaluate if the difference between the estimated altitude of each device and laser altimeter differed from zero within different time intervals during horizontal movements. CatLog did not take consistently take points every 15 seconds, therefore datapoints could not be sorted in 15 second time intervals. Effect size was provided by the ‘lmerTest’ output in R. Significant differences are in bold (α ≤ 0.05).

	15 sec	30 sec	1 min	2 min	3 min	4 min
**AxyAir**						
mean # datapoints	3 ± 0	6± 0	12± 0	24± 0	36± 0	48 ± 0
p-value	**0.01**	0.10	0.29	0.75	0.99	0.97
effect size	-0.81	-0.52	-0.34	-0.10	-5.8 x 10^−3^	-1.2 x 10^−2^
**CatLog**						
mean # datapoints	1± 0	2.25 ± 1.14	3.33 ± 1.44	7.08 ± 2.94	11.17 ± 4.84	15.58 ± 6.88
p-value	NA	**2.20 x 10** ^**−16**^	**5.29 x 10** ^**−12**^	**1.86 x 10** ^**−9**^	**1.91 x 10** ^**−6**^	**4.88 x 10** ^**−4**^
effect size	NA	-3.16	-2.18	-1.90	-1.51	-1.10
**OrniTrack-25**						
mean #datapoints	3 ± 0	6 ± 0	12 ± 0	24 ± 0	36 ± 0	47 ± 3.32
p-value	**2.20 x 10** ^**−16**^	**2.20 x 10** ^**−16**^	**3.65 x 10** ^**−12**^	**5.59 x 10** ^**−9**^	**3.82 x 10** ^**−6**^	**4.88 x 10** ^**−4**^
effect size	-4.52	-3.17	-2.20	-1.84	-1.46	-1.10

The CatLog and OrniTrack-25 connected to 4–8 and 15–18 satellites during each flight, respectively, with minimal variation within a single flight (mean sd = 0.28 satellites for CatLog, mean sd = 0.47 satellites for OrniTrack-25). Due to the lack of variability in the number of satellites, we did not perform a more quantitative assessment of this relationship.

For the CatLog device, PDOP values were only available for the last 7 (out of 10) of the flights due to a change in CatLog units beginning on flight number 4 (as above). Filtering out datapoints with PDOP values >2 removed 3 entire flights from further analysis and an additional 3.5% of data points from remaining flights. When points with PDOP >2 were removed, the mean altitude estimates of remaining points were still much greater than the laser altimeter (S7 Fig) and did not improve overall accuracy, however we were not able to perform a statistical test to assess accuracy due to the low sample size (n = 4) that resulted from removing PDOP values >2. When datapoints with HDOP values >2 were removed from the analysis, this removed 5 entire flights from further analysis for the CatLog device (F25, F27, F28, F29, F35) and 0% of points from remaining flights; it removed no flights or points for the OrniTrack-25 device data. We found that after removing datapoints with HDOP >2 for the CatLog device, estimates were still significantly different from the laser altimeter (p = 0.03). Because no datapoints were removed from the OrniTrack-25 data as all points had HDOP <2, these estimates remained significantly different from the laser altimeter.

We found no significant linear relationship between relative humidity and accuracy of the AxyAir device (Adjusted R^2^ = -0.118; slope = -0.016, p-value = 0.832; S8a Fig). Similarly, we found no significant linear trend between air temperature and accuracy of the AxyAir device (Adjusted R2 = -0.012; slope = -0.13; p-value = 0.373; S8b Fig).

### Vertical movements from 10–60 meters

**Fig 5:**
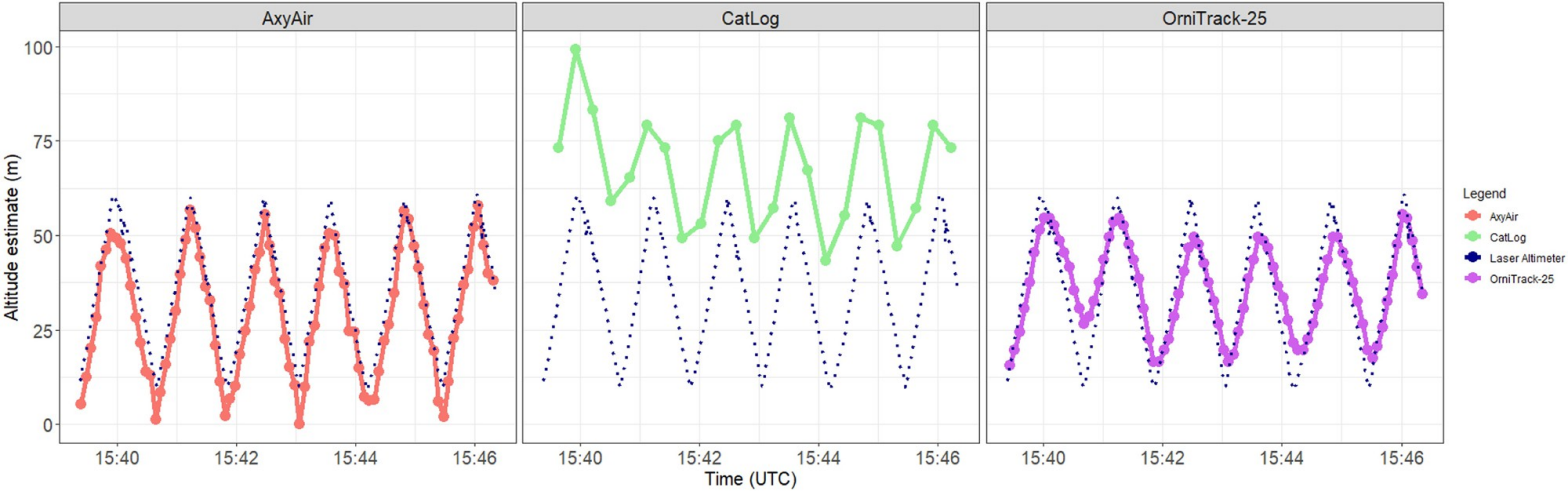
Example of vertical flight movements captured by the laser altimeter (dashed line) and the three different device types on a single flight (flight number F31). On half of the flights (n = 5) the CatLog did not display sinusoidal movements such as the one depicted in this figure, and therefore this represents anomalous movements by the CatLog device.

**Fig 6:**
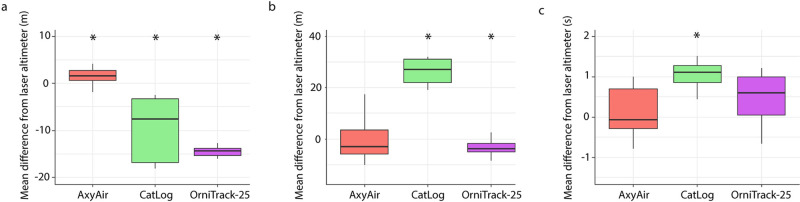
Mean difference between each device and the laser altimeter in a) amplitude (m); b) altitude of wave peak (m); c) wave period (s). Asterisks represent significant differences from the laser altimeter (α ≤ 0.05).

We found that both the AxyAir and OrniTrack-25 consistently captured vertical ascension and descension during sinusoidal vertical movements across all flights (Fig 5), while the CatLog device inconsistently captured vertical sinusoidal movements (e.g. some flights showed sinusoidal movement while others showed no vertical movement at all). For flights in which the CatLog did not capture any sinusoidal movements (n = 5 of 10), we removed these flights from further statistical analysis. All three devices significantly differed from the laser altimeter in their estimate of wave amplitude (S5 Table). However, this difference was much larger for the CatLog and OrniTrack-25 devices, which tended to underestimate amplitude to a much greater extent than the AxyAir device (Fig 6a).

The AxyAir device did not significantly differ from the laser altimeter in its altitude estimate of the wave peak, but generally tended to underestimate the altitude of wave peak by 2.0m (S5 Table; Fig 6b). The OrniTrack-25 device significantly underestimated altitude of wave peak by a mean of 3.2m while the CatLog device significantly over estimated height of wave peak by a mean of 40.40m (S5 Table; Fig 6b). These wave peaks captured by all devices occurred within 11 seconds of the laser altimeter. More specifically, wave peaks occurred on average within 3.9 (± 4.94), 3.52 (± 2.42), and 2.61 (± 2.06) seconds for AxyAir, CatLog, and OrniTrack-25, respectively. Lastly, the AxyAir and OrniTrack-25 devices did not significantly differ from the laser altimeter in their estimate of wave period (AxyAir overestimated by a mean of 0.68 sec; OrniTrack-25 overestimated by a mean of 0.70 sec) while the CatLog significantly overestimated wave period by a mean of 0.95 seconds compared to the laser altimeter (S5 Table; Fig 6c).

All HDOP values for the OrniTrack-25 device on vertical flights were <2, therefore filtering out HDOP values did not impact the accuracy of any of the metrics assessed during vertical flights. For the five flights in which the CatLog device captured vertical sinusoidal movement, filtering out datapoints with HDOP coincided with PDOP values <2 and removed 1 entire flight from analysis (F35) and 0% of data points from remaining flights. Three of the five flights in which sinusoidal movement was not detected had HDOP and PDOP values >2 for all data points on those flights while the remaining two flights had HDOP and PDOP values <2, despite not capturing sinusoidal movements. Because removing data points with HDOP and PDOP values >2 removed entire flights rather than specific data points within a flight and even flights in which sinusoidal movement was not detected had PDOP and HDOP values <2 for all datapoints, this filtering method was not considered to change the accuracy of the CatLog device’s estimates of vertical metrics (wave amplitude, altitude of wave peak, wave period).

## Discussion

Our results show that the AxyAir device, which uses measurements of air pressure to estimate altitude, provided accurate mean altitudes across stationary and horizontal field trials and accurately captured dynamic vertical movements during vertical field trials. The CatLog and OrniTrack-25 devices, which estimate altitude using GPS triangulation, tended to overestimate altitude during horizontal movements and underestimate the amplitude of vertical sinusoidal movements, though errors were much greater in the CatLog device than the OrniTrack-25 device. The relatively low mean error of the OrniTrack-25 device during horizontal and vertical movements suggests that this device may allow sufficient characterization of habitat use in the vertical dimension for some applications (e.g., broad-scale assessments of flight height of birds).

Although we were not able to quantitatively evaluate the relationship between device accuracy and the number of satellites connected to due to limited variability in this metric within each device, we found that OrniTrack-25 generally provided more accurate altitude estimates and connected to a much larger number of satellites than the CatLog device at our study site. It is worth noting, however, that a minimum of 4 satellites are needed to estimate vertical position and the number of satellite connections once this minimum threshold is met may not play a critical role in the accuracy of vertical estimates [47, 48], though further research is warranted to assess the role of this metric across biologging devices.

Unlike previous studies [37, 38], we found that removing datapoints with PDOP values >2 did not improve the accuracy of altitude estimates for the CatLog device. However, given the consistently large error in CatLog altitude estimates, filtering PDOP values might be a feasible method to improve device accuracy in other GPS devices, though more thorough investigation is needed. Similarly, removing datapoints with HDOP values >2 did not improve the altitude estimates of the CatLog and OrniTrack-25 devices. In future studies using DOP values to filter datapoints, the study’s objective resolution of data needed should be carefully considered relative to the accuracy of the altitude estimates when determining which DOP values should be conserved. As an alternative to filtering datapoints based on DOP values, these values can be used as a covariate within the data analysis process as a measurement of potential error, thereby conserving all datapoints collected [29].

We did not observe an impact on the relative humidity or air temperature on the accuracy of AxyAir device altitude estimates and relative humidity or air temperature. However, these factors are important components to consider when using barometric pressure-based devices. Future studies using barometric pressure-based devices to measure altitude in birds should consider and account for these factors to the extent possible. In the case of the AxyAir device used in this study, fine-scale temperature was accounted for when converting pressure values to altitude values (see methods section for further detail). This is an example of one way to account for effects of temperature on barometric pressure readings and, potentially, is one reason why we did not find a strong relationship between the accuracy of the AxyAir and air temperature.

We found an overall improvement in altitude estimates from stationary to moving field trials in both the horizontal and vertical movement trials for the OrniTrack-25 device; mean error decreased from approximately 20.97m during higher flight heights in stationary trials to 6.5m and 3.2m during horizontal and vertical trials, respectively. This was likely due to a reduction in accuracy when a GPS unit is stationary compared to when it is moving [32, 33]. This underscores the importance of assessing location accuracy using data from moving GPS units, since stationary units may result in an inappropriate representation of device accuracy and precision. In additional stationary tests not reported in this study in which both OrniTrack-25 and CatLog devices were anchored on a wooden plank (~12mm height) placed stationary on a rooftop of a known height with no visible aerial obstructions for ~3 hours at a time, we found reduced accuracy and precision of altitude estimates compared to that observed during the horizontal movement tests (CatLog overestimated altitude by a mean of 25.14m ± 42.87; OrniTrack-25 overestimated altitude by a mean of 20.44m ± 47.38). This further suggests that stationary tests may not appropriately capture the accuracy and precision of GPS tracking devices, despite their frequent use inform tracking studies. However, we acknowledge that it may be difficult for other studies to assess device accuracy using a moving UAS, such as the one utilized in this study, due to the monetary cost and labor associated with this method. Therefore, stationary tests may still be useful in broadly assessing the accuracy of device altitude estimates, though we recommend that the resolution of data needed be thoroughly considered prior to drawing conclusions.

Our analysis averaging across different time intervals during horizontal movements suggests that neither the number of GPS points nor the time across which points are averaged improved the flight altitude estimates for devices using GPS triangulation (CatLog, OrniTrack-25). However, we did find that averaging across various time intervals did affect the accuracy of AxyAir altitude estimates. Additional investigation of the horizontal field trial data revealed that a minimum of 5 datapoints (in this study, this equated to averaging over 25 seconds) was required for the AxyAir device to accurately capture the mean flight altitude. In our linear model, we did not find any evidence that GPS device accuracy increased through time (S3 Table). This is an important note for studies considering the use of “GPS burst” settings in which the GPS is turned on for a brief period taking high resolution data. Our results suggest that it cannot be assumed that the last data point in a GPS burst is the most accurate across all devices.

On average, the AxyAir provided accurate mean estimates of sinusoidal movements across two of the metrics evaluated from vertical flights (altitude of wave peak, wave period). Though significantly different from the laser altimeter, the AxyAir estimate of wave amplitude only differed from the laser altimeter by an average of 2.2m and provided the best estimate of wave amplitude across the three devices tested. The OrniTrack-25 device accurately estimated the wave period while the CatLog device significantly overestimated wave period. Though both the OrniTrack-25 and CatLog device accurately captured the time at which wave peaks occurred, estimates of the wave amplitude and altitude of the wave peak from both devices differed significantly from those of the laser altimeter. One potential source of error when estimating the amplitude, the altitude of the wave peak, and the time at which wave peaks occurred is the difference in sampling rates between the laser altimeter and the devices, particularly for the CatLog device in which its inherent coarser resolution likely reduced its ability to capture finer-scale dynamic movements. However, it is unlikely that sampling rate was the predominant driver of error as the AxyAir and OrniTrack-25 devices had equal sampling rates yet exhibited very different errors in amplitude estimation (2.2m for AxyAir; 14.3m for OrniTrack-25). Further, the AxyAir device did not significantly differ from the laser altimeter in its altitude estimate of the wave peak while the OrniTrack-25 device did, despite their equal sampling rates.

It is generally accepted that error in the vertical dimension of a GPS device is equal to approximately three times that of the device’s error in the horizontal dimension [49, 50], attributed to the fact that satellites orbit the Earth at an altitude of approximately three times that of Earth’s radius [51]. According to information provided by the tag manufacturers, this suggests that CatLog and OrniTrack-25 should have vertical errors of approximately 15-30m. Our results from estimates during horizontal movements show that the mean vertical error is often much larger for the CatLog device than this expected range, while the mean vertical error of the OrniTrack 25 device was below the expected range. It is important to note, however, that horizontal positional accuracy (latitude and longitude) was not directly calculated in this study. Errors associated with AxyAir altitude estimates were within the expected error range according to manufacturer specifications (2mbar error ≈ 2.5m under standard atmospheric conditions).

Our observed estimates of device accuracy may assist future studies of bird behavior relative to vertical obstacles such as wind farms. Given the need for accurate flight height estimates for birds to assess collision risk with wind farms both on land and on water, studies using tracking devices to assess flight height should consider the error in altitude estimates from the different tracking devices when planning studies and analyzing results. We found that the pressure-based AxyAir device provides much more accurate estimates of flight height than GPS devices in avian tracking studies. Additionally, the AxyAir device exhibits the capability of sampling at a much finer resolution (up to 1 sample per second) without compromising battery life, unlike the OrniTrack-25 device trialed in this study, which could provide higher resolution data for fine-scale assessments. The CatLog GPS device tested in this study had a vertical error up to 50m in some field trials, which would impede efforts to accurately quantify collision risk based on flight height, as wind turbines range from 75-90m in height [52]. The mean error of the OrniTrack-25 GPS device (6.5m in horizontal field trials; 3.2m in vertical field trials) in this case may provide sufficient accuracy for assessing collision risk depending on the flight heights of the species being evaluated. It is important to note that our objective was to assess the error in altitude estimates across different types of tracking devices and each of the tracking devices assessed here are typically deployed in different study situations; AxyAir devices are archival devices used to assess fine-scale flight behavior over short time periods (days to weeks), CatLog GPS are archival devices used to assess spatial habitat use over short time periods, and OrniTrack devices communicate data over the cellular network and are used to provide information on spatial habitat use over much longer time periods (several months). However, devices that use pressure measurements to assess altitude and communicate data remotely may be feasible in future studies. Thus, the variability in altitude estimate error of different devices may be an important consideration when planning future studies.

Our results also provide useful context for studies focusing on fine-scale flight behavior in birds. For example, many terrestrial birds and seabirds use air thermals for efficient flight and alter their behavior in accordance with air density [53, 54]. Some seabirds perform soaring behaviors and use wind and wave patterns and for efficient flight [55–57]. In these contexts, accurately estimating altitude and dynamic vertical movements is critical to understanding the relationship between environmental variables (like wind) and fine-scale behavior. This continues to be an important subject in avian literature, as wind and atmospheric vertical thermal structure has been and will continue to change under climate change scenarios [58–61]. While our approach of using a drone-mounted laser altimeter did not allow us to assess the accuracy of altitude estimates of bird tracking during natural bird behaviors (e.g., rapid changes in altitude or speed occurring in soaring birds), this approach did allow us to assess broadly how vertical or horizontal movement influenced accuracy in these devices. Our findings suggest that atmospheric pressure-based tags perform well during both vertical and horizontal movement, and can provide sufficient accuracy and resolution to effectively characterize movements such as dynamic soaring arcs of albatrosses, which have a period of approximately 10 seconds [62]. However, studies of finer-scale movements should evaluate the accuracy of altitude measurements relative to the scale of the movements being assessed. Incorporating accuracy estimates of flight height could also improve behavioral modeling (e.g. state space modeling). Increasingly, behavioral models are using relative changes in altitude estimates to assign behavioral states [63, 64] and variability in device accuracy could affect the uncertainty surrounding state classifications within state space models.

It is important to note that this study was conducted in an open field, with minimal aerial obstructions (only potential obstructions were tall pine trees on either side of the field approximately 20-50m from UAS flight locations) and no changes in ground elevation. In actual animal tracking studies, GPS accuracy can further be complicated by obstructions such as degree of vegetative cover [49] or uneven terrain [29]. Similarly, the accuracy of altitude estimates in pressure-based sensors can be influenced by the humidity, temperature, and accuracy of the pressure value for a particular location and time. We accounted for potential variability in environmental conditions between flights by conducting flights on different days and time of day. While we found that barometric pressure-based sensors performed well across a range of temperature and humidity values, during long or distant deployments, changes in baseline atmospheric pressure will need to be corrected using time-and location-specific estimates of local pressure which account for humidity and temperature (e.g., from reanalysis products [65]) to isolate pressure changes that reflect fine-scale changes in altitude from bird movement. As pressure sensors do not provide horizontal locations (e.g. latitude, longitude), scientists may consider using a dual device approach in which both GPS and barometric pressure devices are deployed concurrently, or simultaneously integrated on the same device. However, we found that the OrniTrack-25 device reasonably captured altitude estimates at lower altitudes and accurate wave period estimates during vertical movements, suggesting that using certain GPS devices may be appropriate depending on the study’s objectives. We further suggest maximizing the sampling rate of devices in tracking studies, which increases the ability to capture dynamic flight movements [44]. Specifically, for the AxyAir device, we suggest using a sampling rate that allows altitude at the time scale of interest to be estimated using 5 or more datapoints (AxyAir device can sample up to 1 per second). We were not able to directly assess variability between units of the same device type in this study due to limitations in additional weight added to the UAS and size of the custom-built platform, but this source of variability in the accuracy of altitude estimates could be further studied in future assessments.

As described in Péron et al. (2020), additional sources of error in altitude estimates include error associated with the geoid projection itself, the elevation of terrain, and the GPS horizontal position of the animals, all of which can lead to error in the post-processing calculation of altitude above ground [29]. Furthermore, although elevation above the geoid is approximately equal to MSL, these two metrics may not be perfectly equal and add a further source of error to the altitude estimates upon data transformation. Steep elevation changes in terrain may also be a larger source of error for terrestrial bird studies where investigators may be interested in knowing the altitude above a particular geographical feature, but where both GPS and barometric pressure-based device accuracy may be reduced due to error associated with the geoid projection and sharp changes in atmospheric pressure. However, this may be much less of an issue in studies tracking seabird movements where altitudes are in reference to mean sea level. It is possible the additional post-processing of data, including the use of a state-space model designed to separate error from true bird movements such as the one developed in Péron et al. (2017), may be an option to improve device altitude estimates a posteriori in some cases [64].

Our findings demonstrate that biologging devices using pressure sensors can provide accurate altitude estimates if the pressure sensors are tuned to air pressure changes as in the AxyAir device. To date, many avian tracking studies estimating flight altitude neither account for nor report the error associated with their estimates, complicating efforts to draw conclusions from reported data and compare findings across studies. This work highlights the need to assess study-specific tracking device limitations prior to data collection and to carefully consider and for both scientists and/or tag manufacturers to report the accuracy of vertical height estimates of these tracking devices upon data analysis and reporting.

## Supporting information

S1 Fig Diagram of DJI Phantom 4 Pro+ UAV with custom built platform allowing for concurrent measurements of all three device types during field trials. (TIF)

S2 Fig Diagram of sinusoidal movement metrics quantified during vertical flights. (TIF)

S3 Fig Paired plot of mean altitude estimates for the CatLog device with all HDOP values at 7 different flight heights during stationary movements. Flight heights are shown above each plot in grey.
(TIF)

S4 Fig Paired plot of mean altitude estimates for the OrniTrack-25 device at 7 different flight heights during stationary movements. Flight heights are shown above each plot in grey.
(TIF)

S5 Fig Paired plot of mean altitude estimates of the AxyAir device at 7 different flight heights during stationary movements. Flight heights are shown above each plot in grey.
(TIF)

S6 Fig Paired plot of mean altitude estimates for the CatLog device with points HDOP <2 at 7 different flight heights during stationary movements. Flight heights are shown above each plot in grey.
(TIF)

S7 Fig Paired plot of mean altitude estimates during horizontal movements of the CatLog device with data points with a) all HDOP values; b) HDOP values <2. (TIF)

S8 Fig (TIF)

S1 Table Daily weather conditions recorded during each field trial. (TIF)

S2 Table P-values and effect sizes of paired Wilcoxon signed rank tests evaluating the difference between the estimated flight altitude of each device type and laser altimeter estimate for stationary estimates. Significant differences are shown in bold (α ≤ 0.05).
(TIF)

S3 Table P-values and effect sizes of paired Wilcoxon signed rank tests evaluating the difference between the estimated flight altitude of each device type and laser altimeter estimate for estimates during horizontal movements. Significant differences are shown in bold (α ≤ 0.05).
(TIF)

S4 Table Values for linear mixed effects model with time from start at the fixed effects and flight number as random effect. Numbers correspond to the fixed effect of time since start. Positive slope represents accuracy decreasing over time, negative slope represents accuracy improving over time.
(TIF)

S5 Table P-values and effect sizes of paired Wilcoxon signed rank tests evaluating the difference between the estimated flight altitude of each device type and laser altimeter estimate for estimates during vertical movements. Significant differences are shown in bold (α ≤ 0.05).
(TIF)

S1 Dataset Altitude estimate data for the laser altimeter and all three biologging devices during stationary UAS flight tests. (CSV)

S2 Dataset Altitude estimate data for the laser altimeter and all three biologging devices during horizontal movement UAS flight tests. (CSV)

S3 Dataset Altitude estimate data for the laser altimeter and all three biologging devices during vertical UAS flight tests. (CSV)
