# Supplementary figures and images for: Assessing the accuracy of altitude estimates in avian biologging devices
Source: PLoS One. 2022 Oct 26;17(10):e0276098. doi: 10.1371/journal.pone.0276098 (PMC9605028; doi:10.1371/journal.pone.0276098)

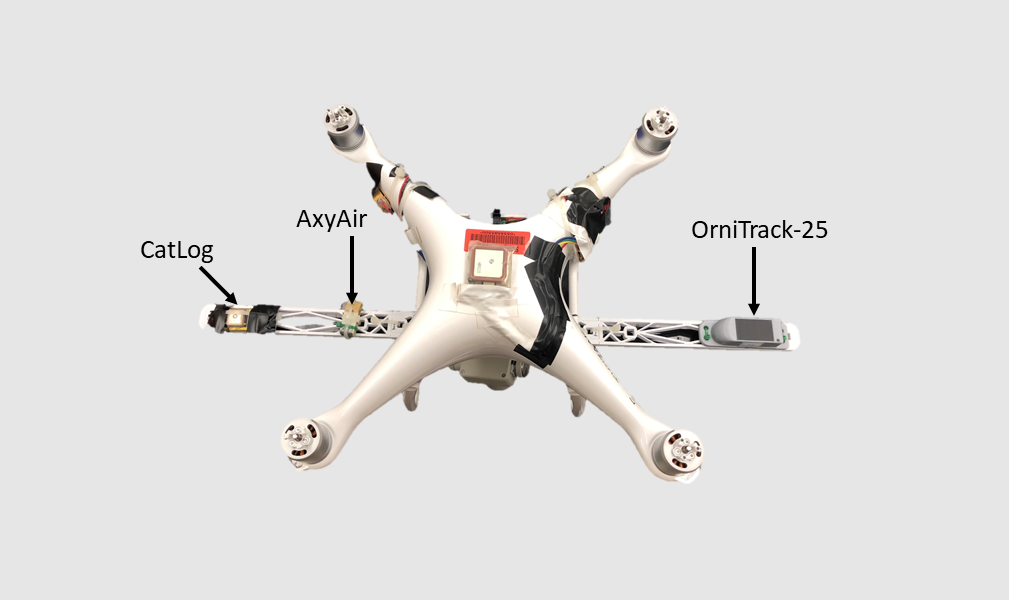

Supplement: S1 Fig — (TIF) [file pone.0276098.s001.tif]

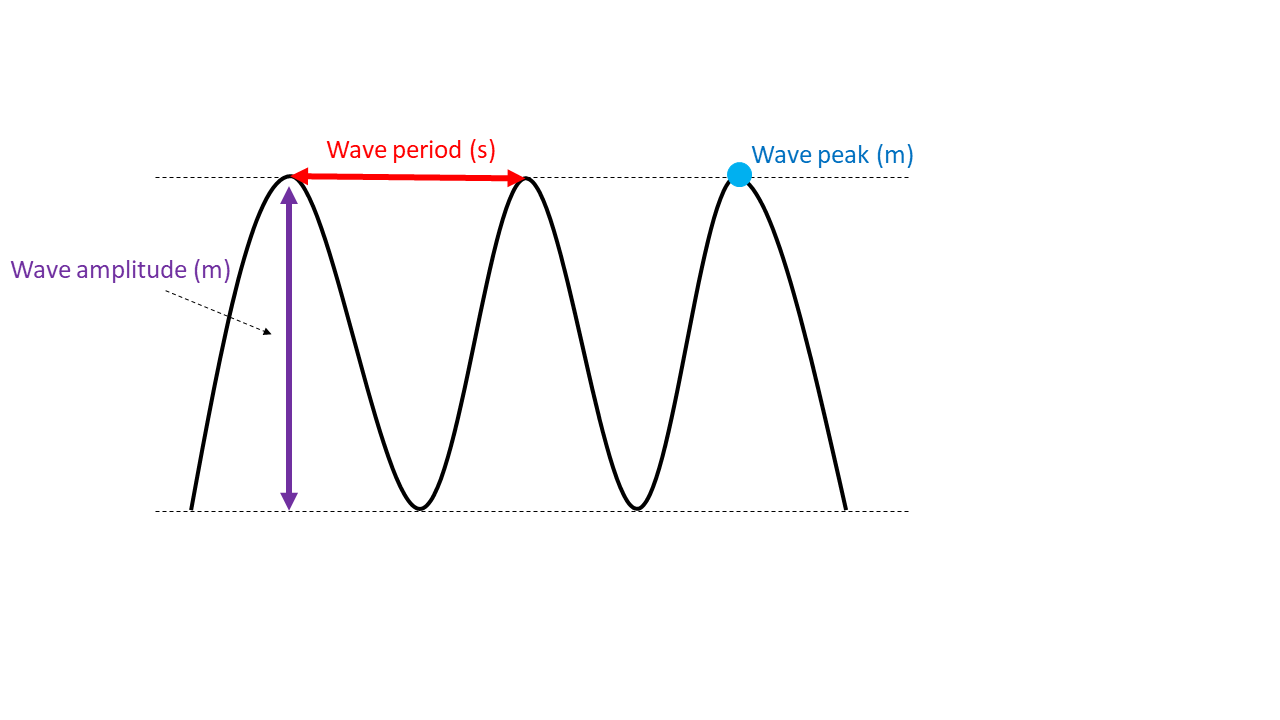

Supplement: S2 Fig — (TIF) [file pone.0276098.s002.tif]

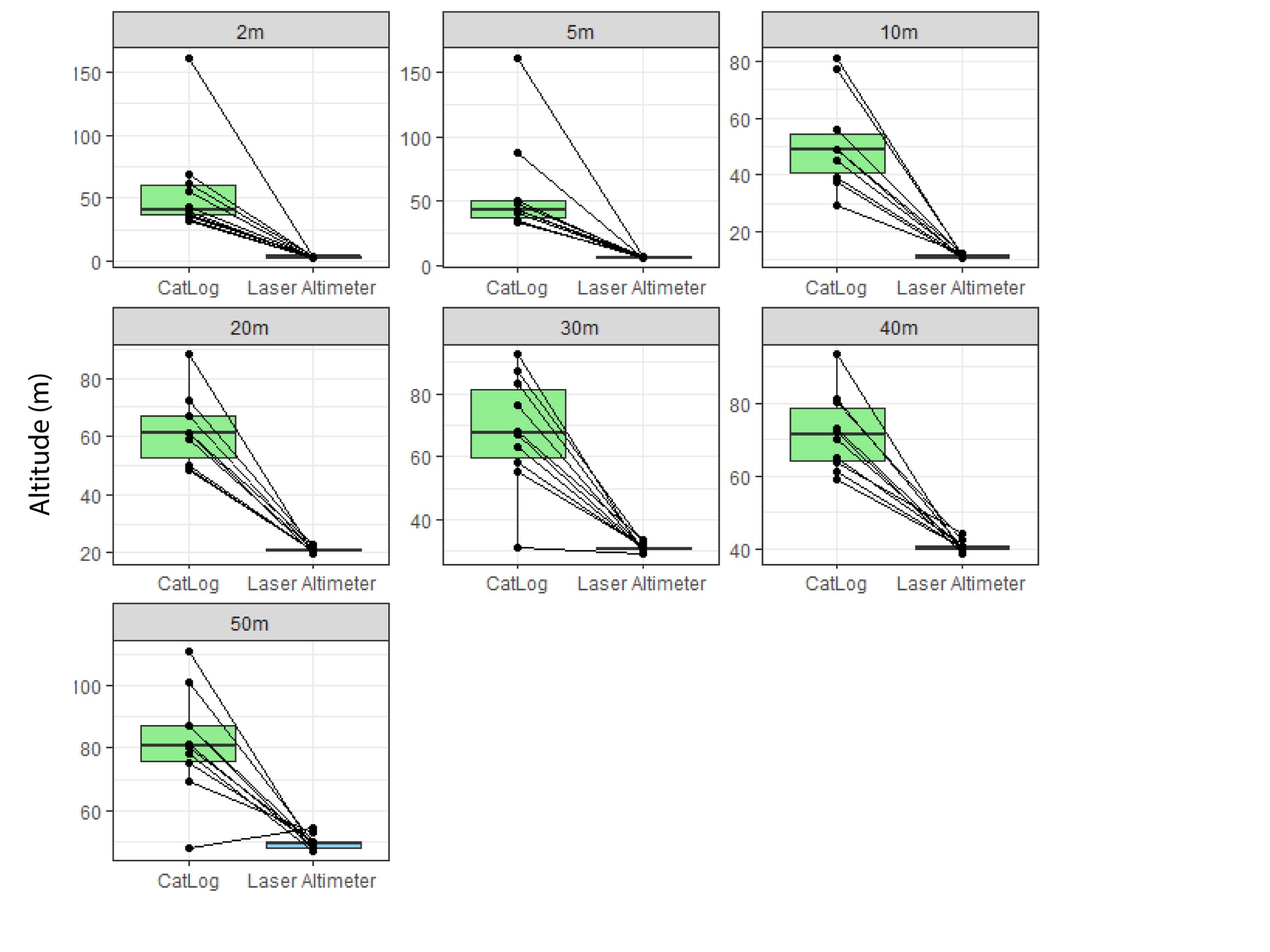

Supplement: S3 Fig — Flight heights are shown above each plot in grey. (TIF) [file pone.0276098.s003.tif]

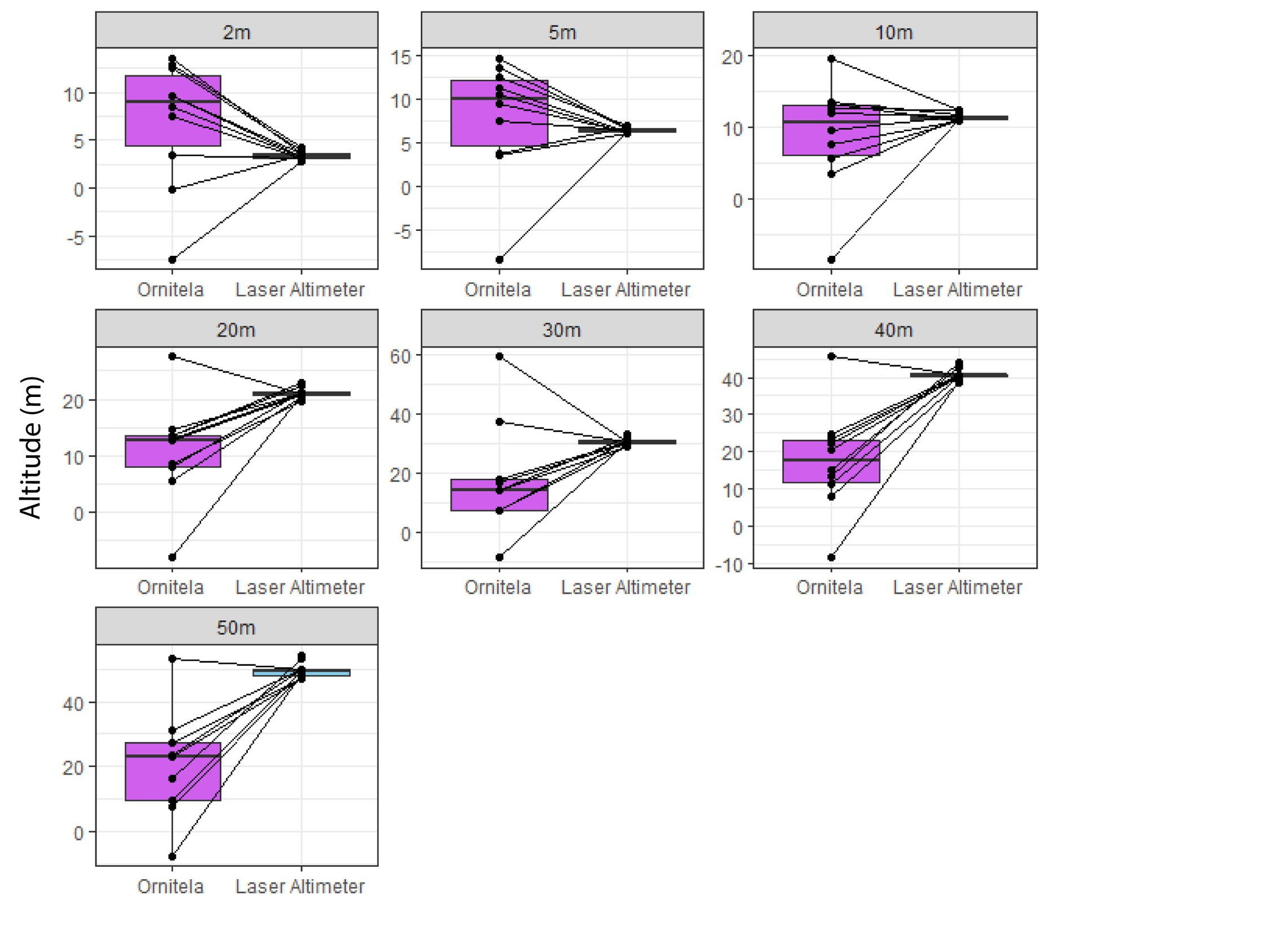

Supplement: S4 Fig — Flight heights are shown above each plot in grey. (TIF) [file pone.0276098.s004.tif]

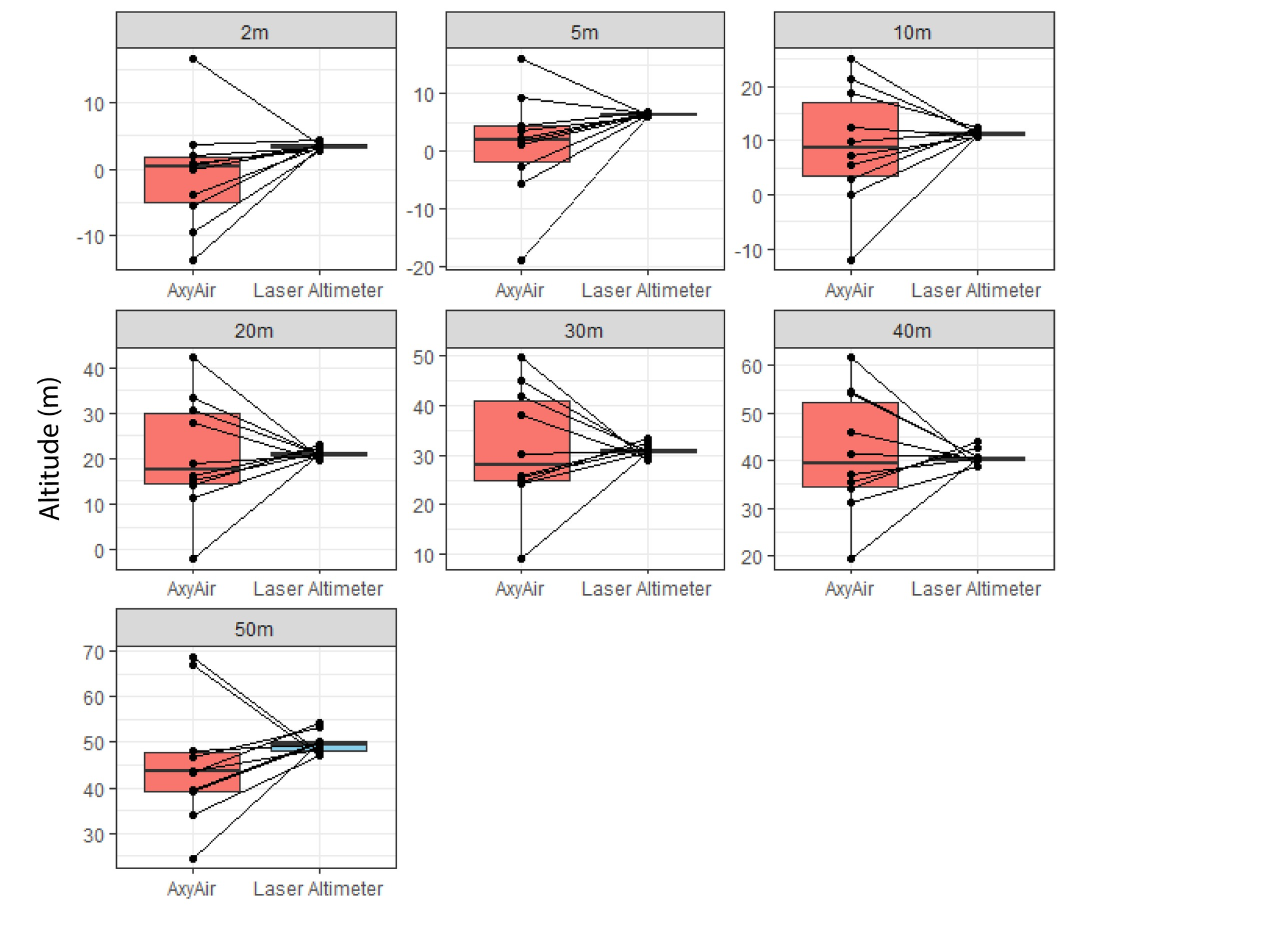

Supplement: S5 Fig — Flight heights are shown above each plot in grey. (TIF) [file pone.0276098.s005.tif]

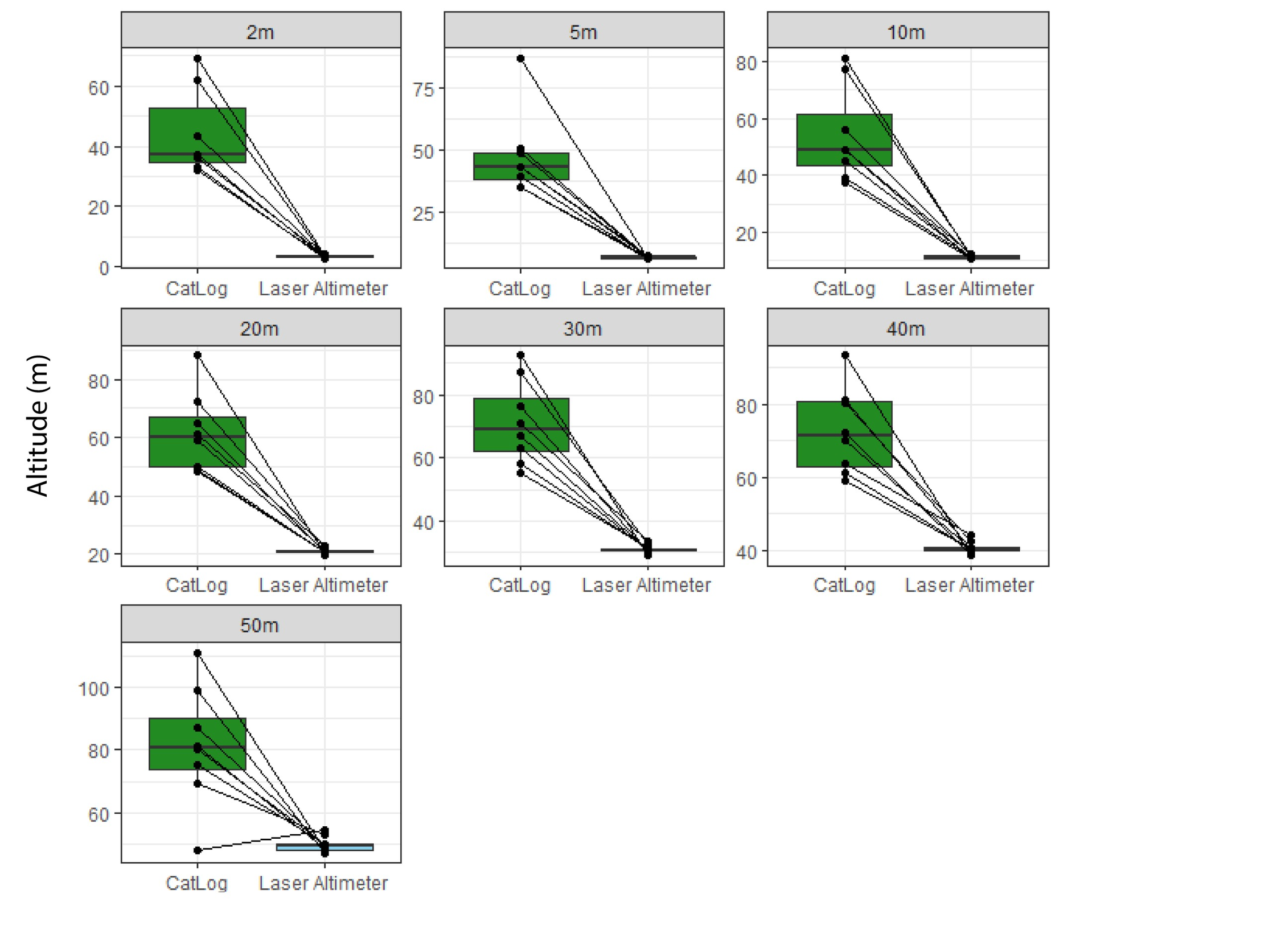

Supplement: S6 Fig — Flight heights are shown above each plot in grey. (TIF) [file pone.0276098.s006.tif]

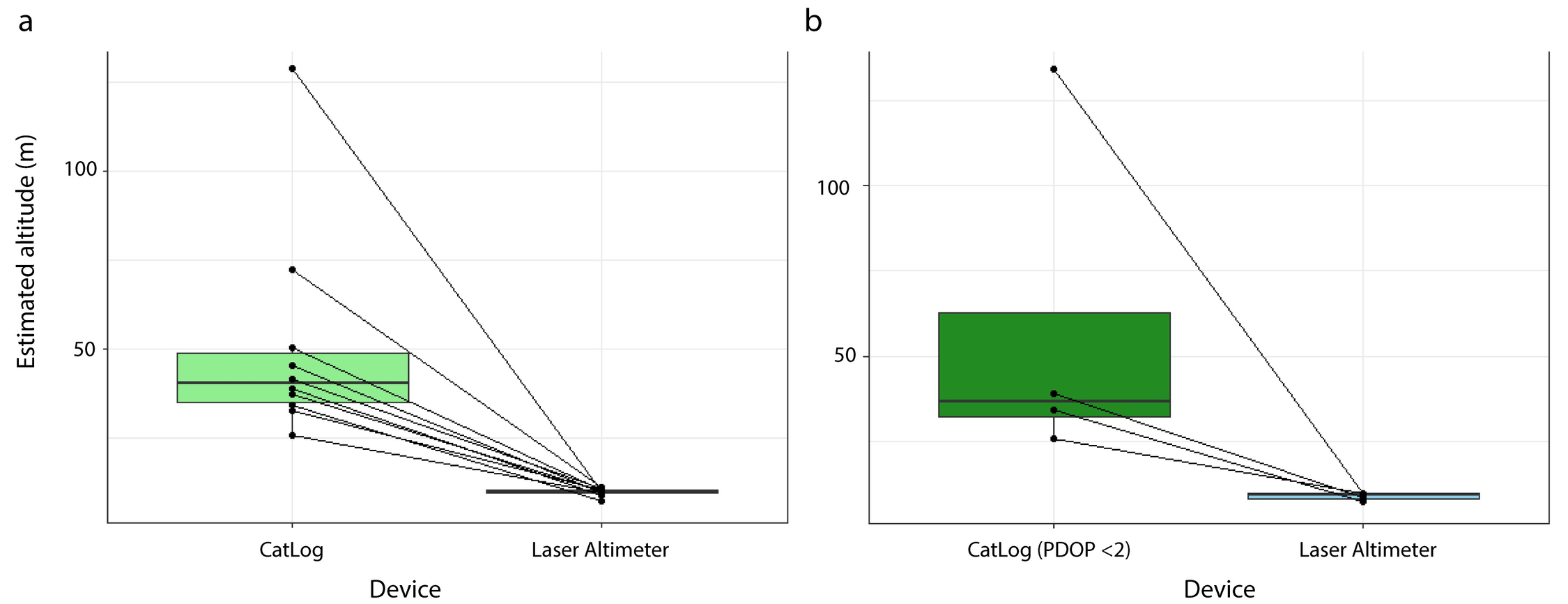

Supplement: S7 Fig — (TIF) [file pone.0276098.s007.tif]

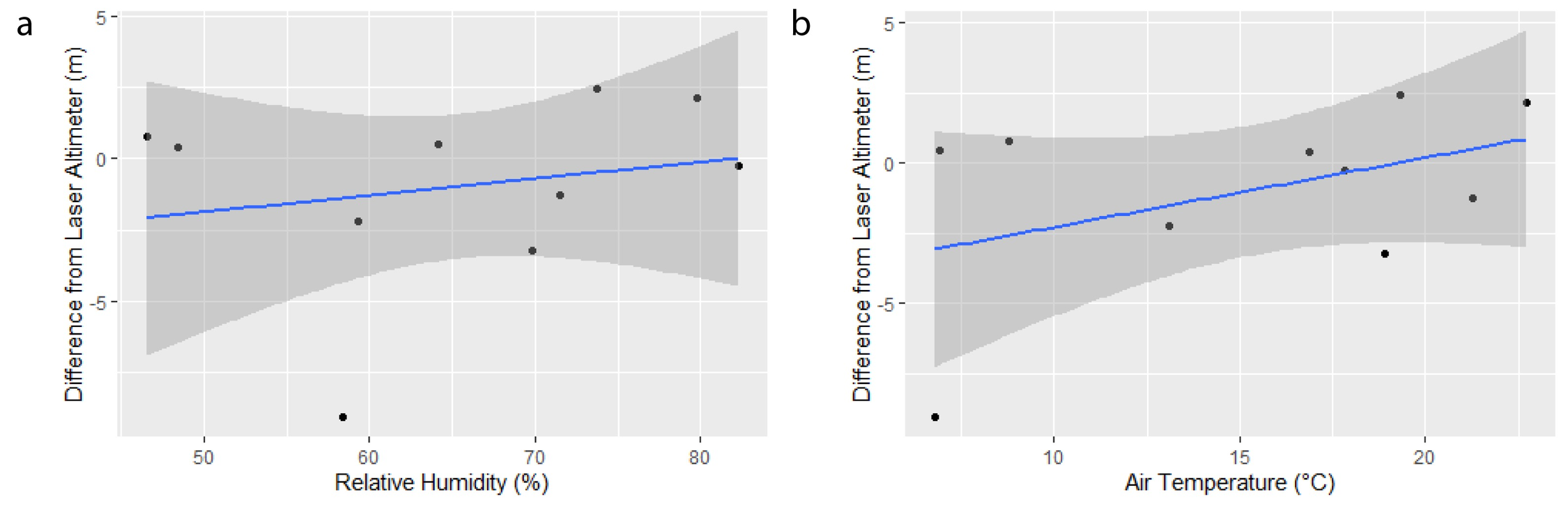

Supplement: S8 Fig — (TIF) [file pone.0276098.s008.tif]

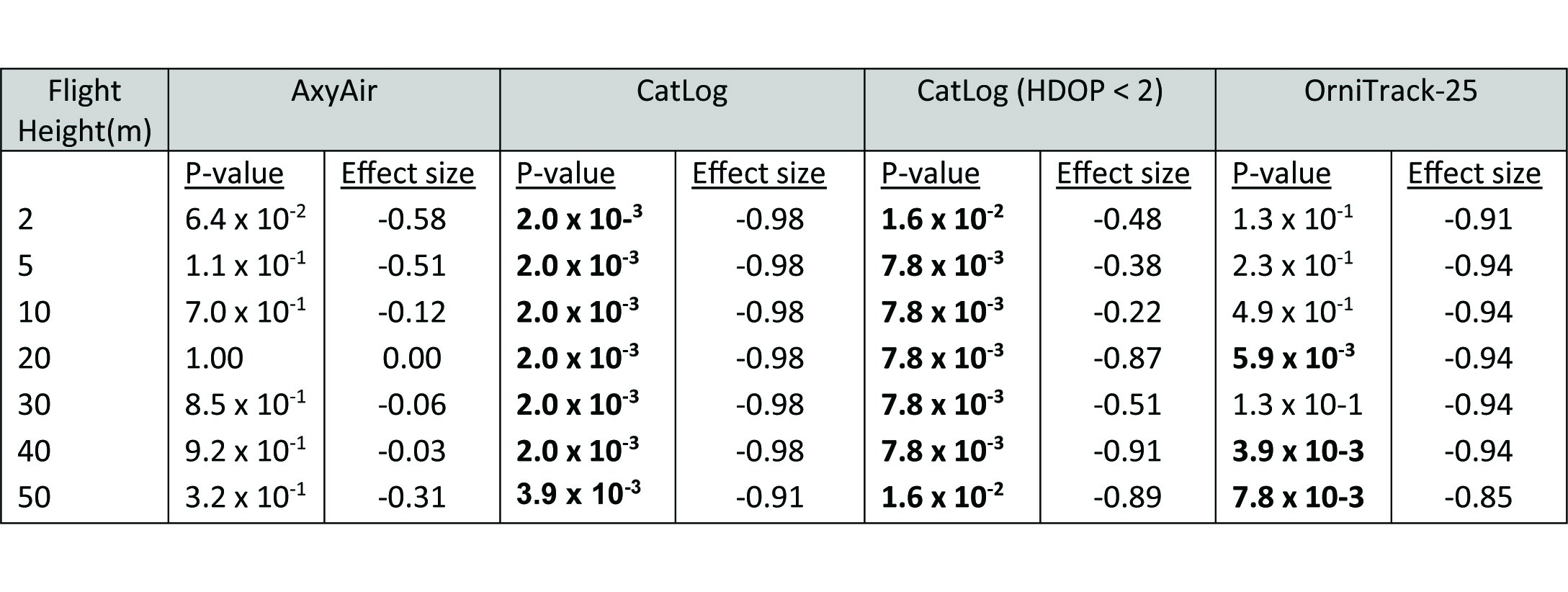

Supplement: S1 Table — (TIF) [file pone.0276098.s009.tif]

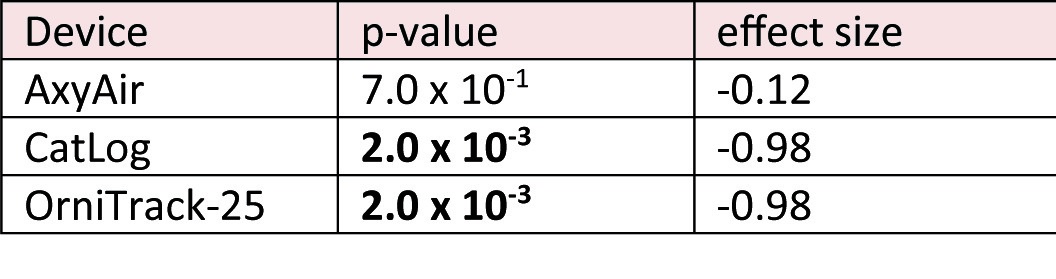

Supplement: S2 Table — Significant differences are shown in bold (α ≤ 0.05). (TIF) [file pone.0276098.s010.tif]

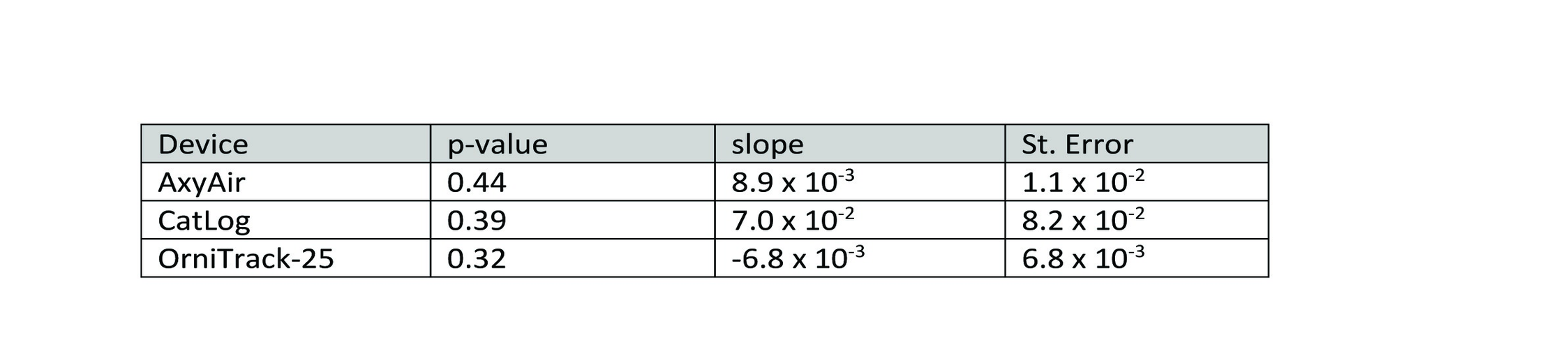

Supplement: S3 Table — Significant differences are shown in bold (α ≤ 0.05). (TIF) [file pone.0276098.s011.tif]

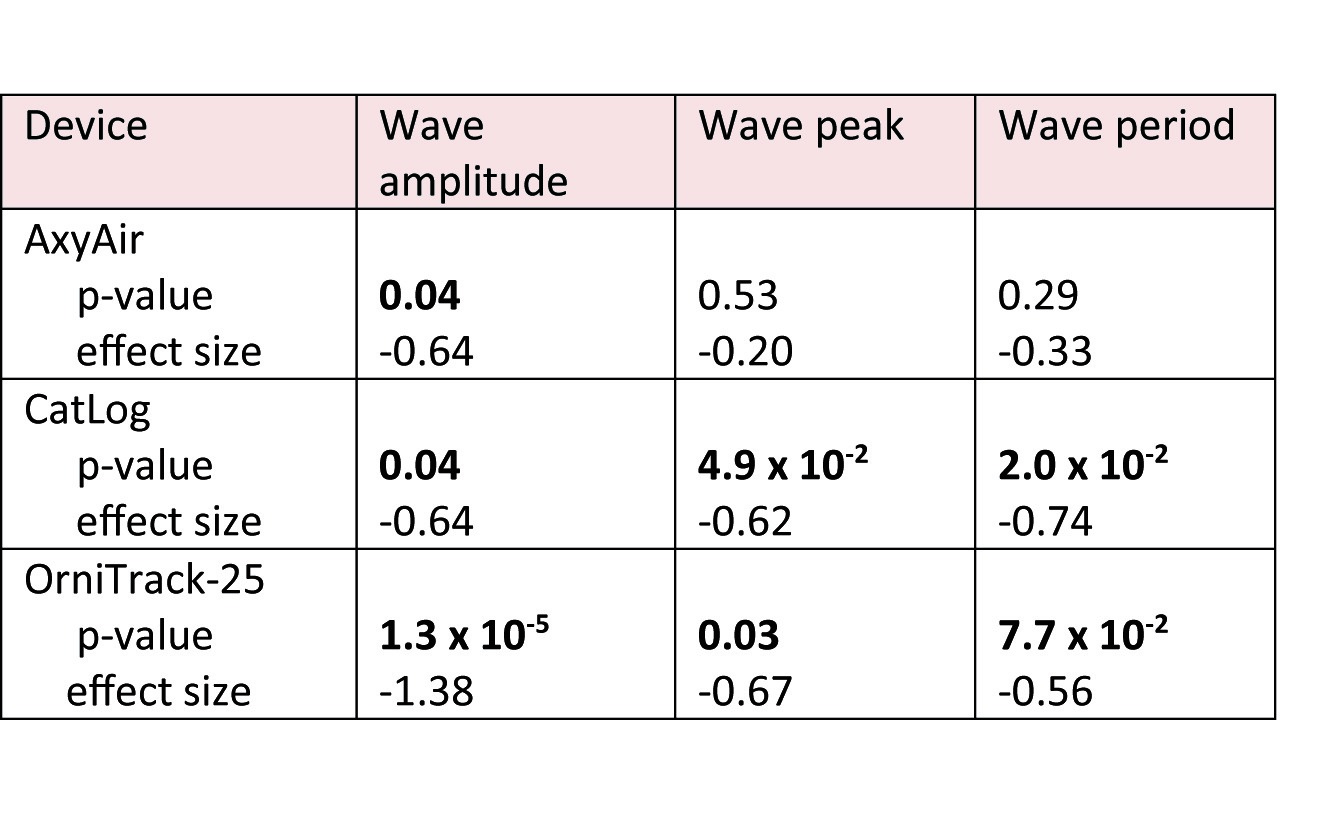

Supplement: S4 Table — Numbers correspond to the fixed effect of time since start. Positive slope represents accuracy decreasing over time, negative slope represents accuracy improving over time. (TIF) [file pone.0276098.s012.tif]

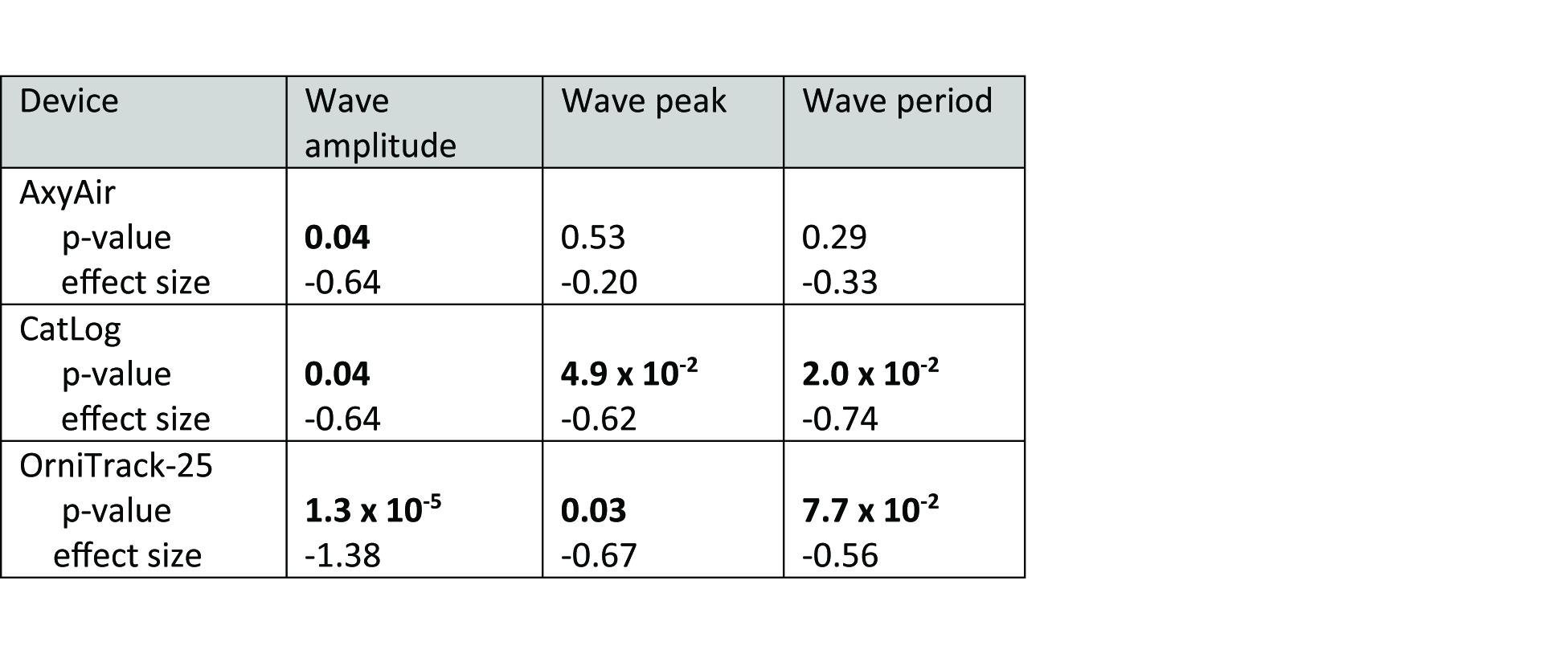

Supplement: S5 Table — Significant differences are shown in bold (α ≤ 0.05). (TIF) [file pone.0276098.s013.tif]
